# EsGLUT4 and CHHBP are involved in the regulation of glucose homeostasis in the crustacean *Eriocheir sinensis*

**DOI:** 10.1242/bio.027532

**Published:** 2017-07-27

**Authors:** Ran Li, Jin-Ze Tian, Mo-Ran Wang, Li-Na Zhu, Jin-Sheng Sun

**Affiliations:** 1 Tianjin Key Laboratory of Animal and Plant Resistance, College of Life Science, Tianjin Normal University, Tianjin 300387, People's Republic of China; 2 Tianjin Key Laboratory of Aqua-ecology and Aquaculture, Department of Fisheries Science, Tianjin Agricultural University, Tianjin 300384, People's Republic of China; 3 Tianjin Center for Control and Prevention of Aquatic Animal Infectious Disease, Tianjin 300221, People's Republic of China

**Keywords:** Glucose transporter 4 (GLUT4), Crustacean hyperglycemic hormone (CHH), CHH binding protein (CHHBP), Protein-protein interactions, *Eriocheir sinensis*

## Abstract

Glucose is an essential energy source for both vertebrates and invertebrates. In mammals, glucose uptake is mediated primarily by glucose transporters (GLUTs), members of the major facilitator superfamily (MFS) of passive transporters. Among the GLUTs, GLUT4 is the main glucose transporter in muscles and adipocytes. In skeletal muscle cells, GLUT4 interacts with the lipid raft protein flotillin to transport glucose upon stimulation by insulin. Although several studies have examined GLUT4 function in mammals, few have been performed in crustaceans, which also use glucose as their main energy source. Crustacean hyperglycemic hormone (CHH) is a multifunctional neurohormone found only in arthropods, and one of its roles is to regulate glucose homeostasis. However, the molecular mechanism that underlies CHH regulation and whether GLUT4 is involved in its regulation in crustaceans remain unclear. In the present study, we identified a full-length GLUT4 cDNA sequence (defined herein as EsGLUT4) from the Chinese mitten crab *Eriocheir sinensis* and analyzed its tissue distribution and cellular localization. By the ForteBio Octet system, two large hydrophilic regions within EsGLUT4 were found to interact with the CHH binding protein (CHHBP), an *E. sinensis* flotillin-like protein. Interestingly, live-cell imaging indicated that EsGLUT4 and CHHBP responded simultaneously upon stimulation by CHH, resulting in glucose release. In contrast to insulin-dependent GLUT4, however, EsGLUT4 and CHHBP were present within cytoplasmic vesicles, both translocating to the plasma membrane upon CHH stimulation. In conclusion, our results provide new evidence for the involvement of EsGLUT4 and CHHBP in the regulation of glucose homeostasis in crustacean carbohydrate metabolism.

## Abstract (precis)

**Summary:** Here we identified that Glucose transporter 4 (GLUT4) could interact with CHH binding protein (CHHBP) to regulate CHH-stimulated glucose release in *Eriocheir sinensis*.

## INTRODUCTION

Nutrient absorption is critical for the growth, reproduction and well-being of animals, and glucose absorption and utilization is especially important. In mammals, the uptake of glucose into cells is mediated mainly by sodium-coupled transporters (SGLTs) and glucose transporters (GLUTs) (Sala-Rabanal et al., 2016; Scheepers et al., 2004). SGLTs are secondary active or sodium-coupled transporters, which couple glucose influx to sodium influx down the sodium electrochemical potential gradient across the plasma membrane (Sala-Rabanal et al., 2016). By contrast, GLUTs are members of the major facilitator superfamily (MFS) of passive transporters, and they allow the facilitative diffusion of glucose and other hexoses across the plasma membrane (Mueckler, 1994; Sun et al., 2012). Among the GLUTs, GLUT1-4 have been described thus far because of their participation in fundamental physiological and pathophysiological processes (Mueckler, 1994). GLUT1 plays a special role in the uptake of glucose into erythrocytes and transporting glucose across the blood-brain barrier (Klepper, 2008). GLUT2 acts as a high-capacity transport system to allow the uninhibited flux of glucose into or out of cells (Santer et al., 1997). GLUT3 is the main neuronal glucose transporter which uptakes glucose into neurons (Simpson et al., 2008). GLUT4 plays an important role in regulating glucose transport in muscles and adipocytes, and it functions as a typical facilitative glucose transporter in the uptake of glucose into fat and muscle cells in response to insulin (Abel et al., 2001; Bryant et al., 2002; James et al., 1988, 1989). Under basal conditions, the majority of GLUT4 is localized in storage vesicles in the cytoplasm of skeletal muscle cells (Fecchi et al., 2006). When an insulin receptor on the cell surface is activated by insulin, downstream signals induce a rapid increase in glucose uptake by inducing GLUT4 translocation from the storage vesicles to the plasma membrane (Dugani and Klip, 2005; Luiken et al., 2015; Malide et al., 2000; Slot et al., 1991). Therefore, the insulin-dependent transient translocation of GLUT4 plays an important role in the physiological metabolism in mammals.

GLUT4 translocation in mammals is involved in the Cbl/C3G/TC10 (casitas B-cell lymphoma/cyanidin-3-O-glucoside/a small GTP-binding protein) signaling pathway (Baumann et al., 2000; Chiang et al., 2001; Kimura et al., 2001). Under stimulation by insulin, this pathway signals the Cbl-CAP (Cbl associated protein) complex to bind flotillin-1 in lipid rafts, leading to the generation of a signal that is crucial for glucose uptake (Baumann et al., 2000; Kanzaki, 2006; Lin et al., 2001; Ribon and Saltiel, 1997). Flotillin-1, a member of the flotillin family, was originally identified as a membrane protein residing in cholesterol-enriched domains that play an important role in glucose uptake in response to insulin signaling (Bauer and Pelkmans, 2006; Bickel et al., 1997; Salzer and Prohaska, 2001). Fecchi et al. (2006) reported that flotillin-1 colocalizes with GLUT4 in the perinuclear stores of skeletal muscle cells in the absence of insulin. After a brief stimulatory period by insulin, GLUT4 forms a stable complex with flotillin-1. Then GLUT4 moves together with flotillin-1 from intracellular stores to the plasma membrane through the PI3K- and PKCζ-dependent pathway. Disruption of flotillin-1 domains results in GLUT4 movement and the glucose uptake to be blocked. Thus, the binding of GLUT4 to flotillin-1 is the key step in GLUT4 translocation from the cytoplasm to the plasma membrane in response to insulin.

Although several studies have examined GLUT4 function upon stimulation by insulin in mammals, few have been performed in crustaceans. In vertebrates, blood glucose concentration is maintained to a certain level under the combined action of insulin and glucagon. In crustaceans, some reports indicate that insulin or insulin-like peptides exist, but the hemolymph glucose levels are elevated in response to stress, under the control of crustacean hyperglycemic hormone (CHH). Unlike glucagon secreted from pancreatic cells in vertebrates, CHH is the primary neuroendocrine hormone that regulates glucose homeostasis throughout the crustacean life cycle (Fanjul-Moles, 2006). CHH is synthesized and secreted from the X-organ/sinus gland (XO/SG) complex in the crustacean eyestalk (Hsu et al., 2006; Wanlem et al., 2011), and it plays crucial roles in many biological processes such as lipid and carbohydrate metabolism, osmoregulation, the immune response, molting, vitellogenesis, and the stress response (Chung and Webster, 2003; Fanjul-Moles, 2006; Liu et al., 2014; Serrano et al., 2003). Moreover, CHH is the main hormone that can elevate the glucose level in crustaceans to date. Thus, it regulates carbohydrate metabolic pathways (Santos and Keller, 1993). The hepatopancreas and muscle, the major metabolic sites that mediate energy metabolism, are the primary target tissues of CHH (Chung et al., 2010; Katayama and Chung, 2009; Kummer and Keller, 1993; Webster et al., 2012). Furthermore, CHH regulates glycogen synthesis and degradation to maintain glucose homeostasis in these tissues. Eyestalk ablation, which is the common method used to eliminate the effects of CHH, results in the activation of glycogen synthase (GS) and in the simultaneous inactivation of glycogen phosphorylase (GP). Injection of CHH decreases GS activity and results in GP activation, subsequently inhibiting the conversion of glucose into glycogen in the hepatopancreas (Sedlmeier, 1982). Therefore, CHH plays a regulatory role in the carbohydrate metabolic pathways of crustaceans.

Although a number of studies reported that CHH regulates GP and GS to maintain glucose homeostasis, the role of GLUT4 in CHH stimulation, which has an equally important role in the regulation of the glucose level, has not yet been characterized in crustaceans. CHH has a very short half-life (*T1/2*) of 5–10 min. Besides, under treatment of stressors, its release raises the glucose level of hemolymphs within 30 min, which lasts for 2–3 h (Chung et al., 2010; Chung and Webster, 2005, 2008; Webster, 1996). We previously identified CHH binding protein (CHHBP), a flotillin-like protein, as a putative receptor of CHH in *Eriocheir sinensis* (Li et al., 2016). The recombinant *E. sinensis* CHH (defined herein as EsCHH) was expressed and injected into *E. sinensis in vitro*, resulting in a significant increase in the blood glucose level at 2 h after CHH injection (Li et al., 2016). However, it is not known whether GLUT4 participates in the transient increase in the blood glucose level induced by CHH during this period in *E. sinensis*. In the present study, we cloned a *GLUT4* cDNA from *E. sinensis* (defined herein as *EsGLUT4*). We investigated the cellular localization of EsGLUT4 and analyzed the interaction between EsGLUT4 and CHHBP using biomolecular assays. We demonstrated that EsGLUT4 might form a protein complex with CHHBP to regulate CHH-stimulated glucose release in *E. sinensis*. Furthermore, we also showed the trafficking of EsGLUT4 and CHHBP upon stimulation by CHH, which has not been described in crustaceans.

## RESULTS

### Cloning and sequence analysis of EsGLUT4

**Fig. 1.**
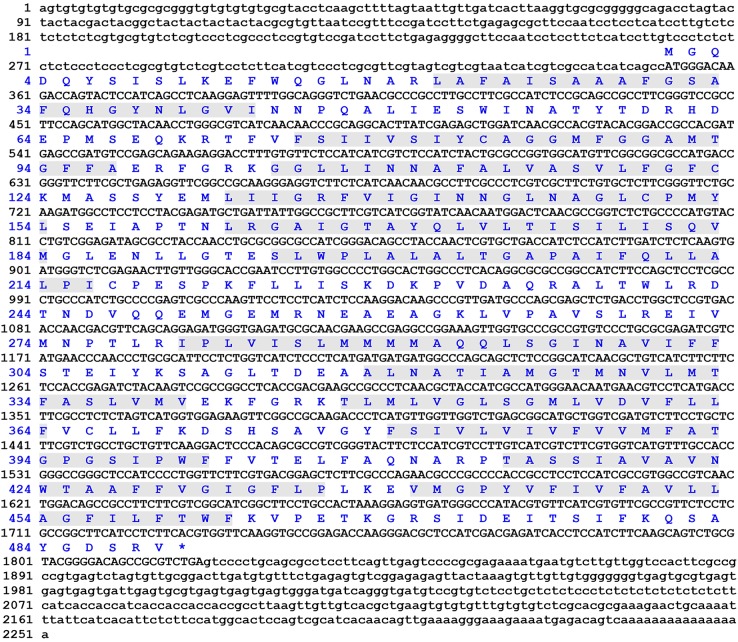
**Deduced amino acid sequence of EsGLUT4 from *E. sinensis.*** The gray-shaded areas represent the twelve transmembrane regions*.* The stop codon is marked with an asterisk.

Gene-specific primers targeting the 5′- and 3′-untranslated regions of GLUT4 were designed based on the transcriptome of the Chinese mitten crab (GLUT4 5′ F/R and GLUT4 3′ F/R, Table S1). Using nested PCR, we cloned a GLUT4 cDNA, designated herein as *E. sinensis GLUT4* (EsGLUT4), from the hepatopancreas of *E. sinensis*. The entire EsGLUT4 cDNA contained a 351 bp 5′-untranslated sequence, a 1470 bp open reading frame (ORF), and a 430 bp 3′-untranslated sequence (Fig. 1). The ORF encoded a polypeptide of 489 amino acids with an isoelectric point of 6.03 and a predicted molecular weight of 53 kDa. BLASTp analysis showed that EsGLUT4 possessed 12 transmembrane regions typical of the sugar transporter subfamily of the MFS, which is one of the largest superfamilies of ubiquitously expressed secondary transporters (Deng et al., 2014; Pao et al., 1998). This cDNA sequence, which has not been previously reported or characterized, was deposited into the GenBank database under accession number KT963013.

### Phylogenetic analysis of EsGLUT4

**Fig. 2.**
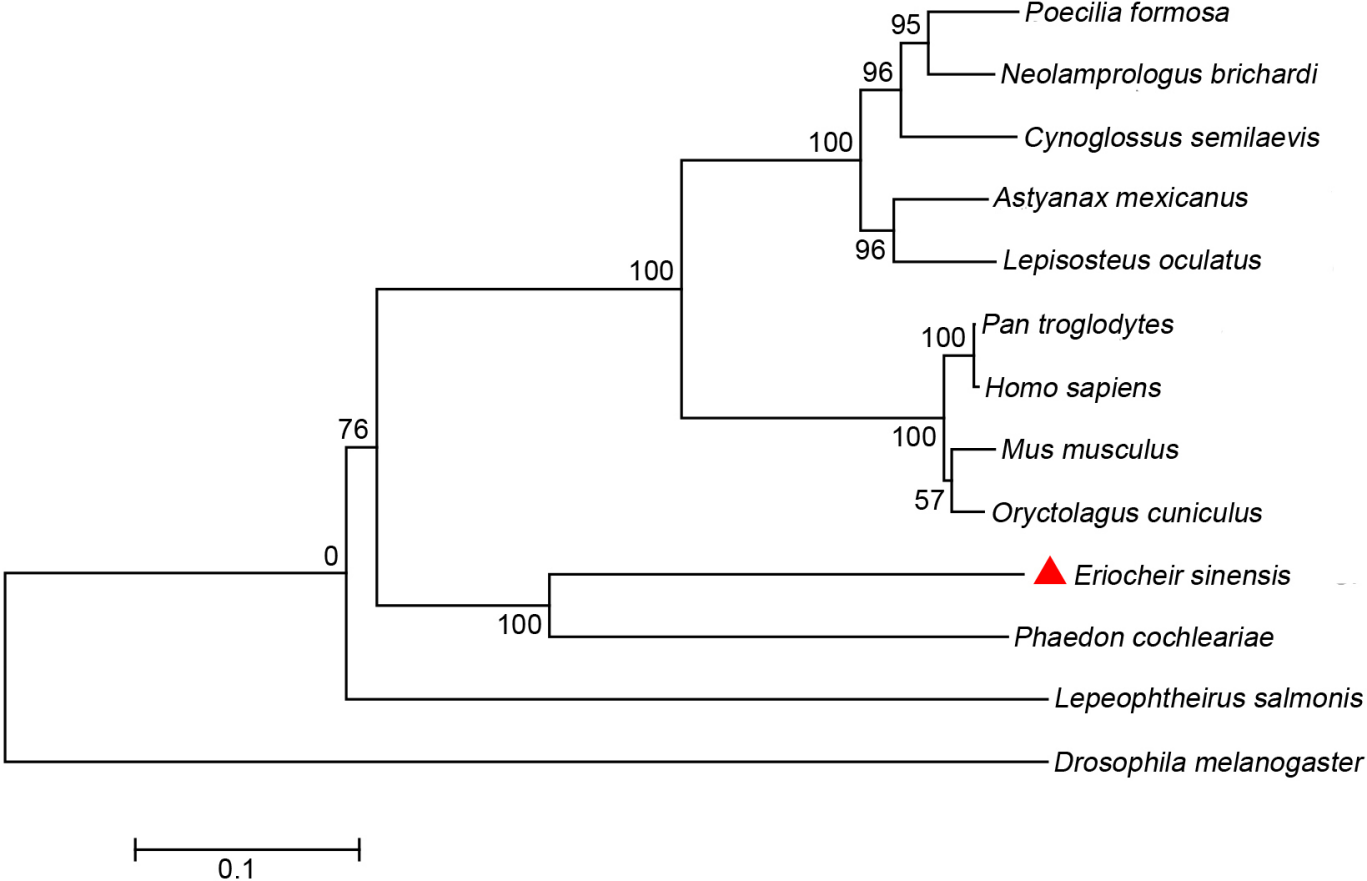
**Phylogenetic tree of EsGLUT4 from crustaceans, other invertebrates, and vertebrates.** The tree was constructed using the neighbor-joining algorithm in Mega 4.0 software, which was based on the alignment of multiple sequences in ClustalW. Bootstrap values of 1000 replicates (%) are indicated for the branches. The bar (0.1) indicates the genetic distance. The GLUT4 proteins included in the phylogenetic analysis were as follows: human (*Homo sapiens*: M91463.1), mustard beetle (*Phaedon cochleariae*: AHF27418.1), chimpanzee (*Pan troglodytes*: XM_001169794.3), hare (*Oryctolagus cuniculus*: AY339876.1), rat (*Mus musculus*: NM_009204.2), tongue sole (*Cynoglossus semilaevis*: XM_008321766.1), Amazon molly (*Poecilia formosa*: XM_007551253.1), fairy cichlid (*Neolamprologus brichardi*: XM_006807697.1), spotted gar (*Lepisosteus oculatus*: XM_006627433.1), Mexican tetra (*Astyanax mexicanus*: XM_007255725.1), fruit fly (*Drosophila melanogaster*: EU312164.1), and salmon louse (*Lepeophtheirus salmonis*: BT077482.1).

To study the molecular evolutionary relationships of EsGLUT4, a phylogenetic tree was constructed using the neighbor-joining method with 1000 bootstraps based on the multiple alignments of GLUT4 from crustaceans, other invertebrates, and vertebrates. As shown in Fig. 2, EsGLUT4 clustered together with GLUT4 from a beetle belonging to Arthropoda (*Phaedon cochleariae*; GenBank accession no. AHF27418.1). BLASTp analysis revealed that the EsGLUT4 protein shared 58% sequence identity with the GLUT4 protein of *Phaedon cochleariae*, indicating that EsGLUT4 was highly homologous with GLUT4 in arthropoda. Additionally, EsGLUT4 formed a sister group with GLUT4 proteins of the vertebrate class Osteichthyes that included tongue sole (*Cynoglossus semilaevis*: XM_008321766.1), Amazon molly (*Poecilia formosa*: XM_007551253.1), fairy cichlid (*Neolamprologus brichardi*: XM_006807697.1), spotted gar (*Lepisosteus oculatus*: XM_006627433.1), and Mexican tetra (*Astyanax mexicanus*: XM_007255725.1).

### Expression and purification of rEsCHH, rEsGLUT4 and rCHHBP proteins

**Fig. 3.**
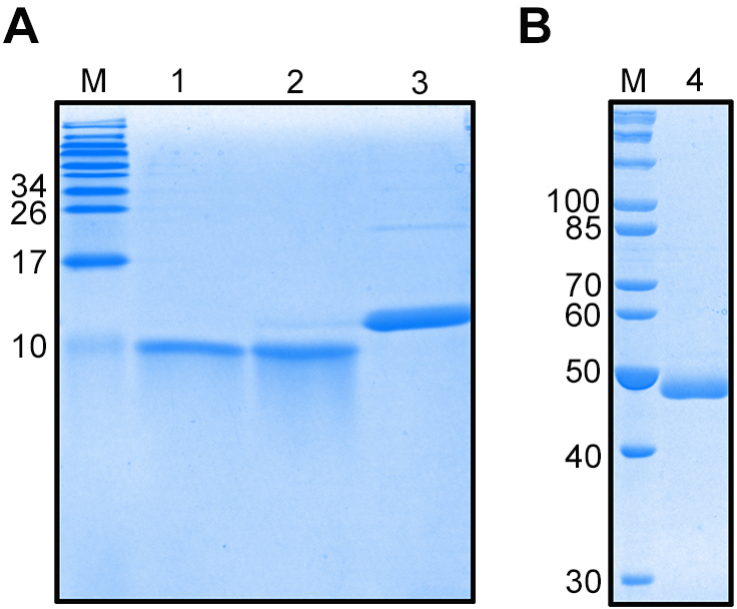
**Expression and purification of the extracellular and intracellular regions of recombinant CHH, EsGLUT4 and the CHHBP protein.** The purified recombinant proteins were subjected to SDS-PAGE and stained with Coomassie Brilliant Blue R-250. Lane M: protein molecular weight standard. Lane 1: CHH protein. Lane 2: extracellular region of EsGLUT4. Lane 3: intracellular region of EsGLUT4. Lane 4: CHHBP protein.

The two main hydrophilic regions within EsGLUT4, as well as CHH and CHHBP, were expressed in *Escherichia coli* Rosetta-gami (DE3) cells. The purified recombinant proteins were then subjected to SDS-PAGE. As shown in Fig. 3, the apparent sizes of purified CHH protein, purified extracellular region of EsGLUT4, and purified intracellular region of EsGLUT4 were 10 kDa, 8.9 kDa, and 11.9 kDa, respectively (Fig. 3A), and purified CHHBP protein had an apparent size of 48 kDa (Fig. 3B). Using the prokaryotic expression system, the rCHH protein was expressed with a His-tag, the two rEsGLUT4 regions were expressed as fusion proteins with His- and T7-tags, and the rCHHBP protein was expressed as a fusion protein with a His-tag and an S-tag, which increased their predicted molecular weights. The purified intracellular and extracellular regions of the rEsGLUT4 protein were verified by SDS-PAGE (12%). The target bands were excised from the gel with a sterile scalpel and digested with trypsin. Then the digested samples were analyzed using an advanced MALDI-TOF/TOF mass spectrometer UltrafleXtrem (Bruke, Germany). The identified peptide fragments matched the deduced amino acid sequences of the intracellular and extracellular regions of rEsGLUT4 (data not shown).

### Use of the Octet RED96^®^ biosensor to analyze the binding affinity between CHHBP and EsGLUT4

**Fig. 4.**
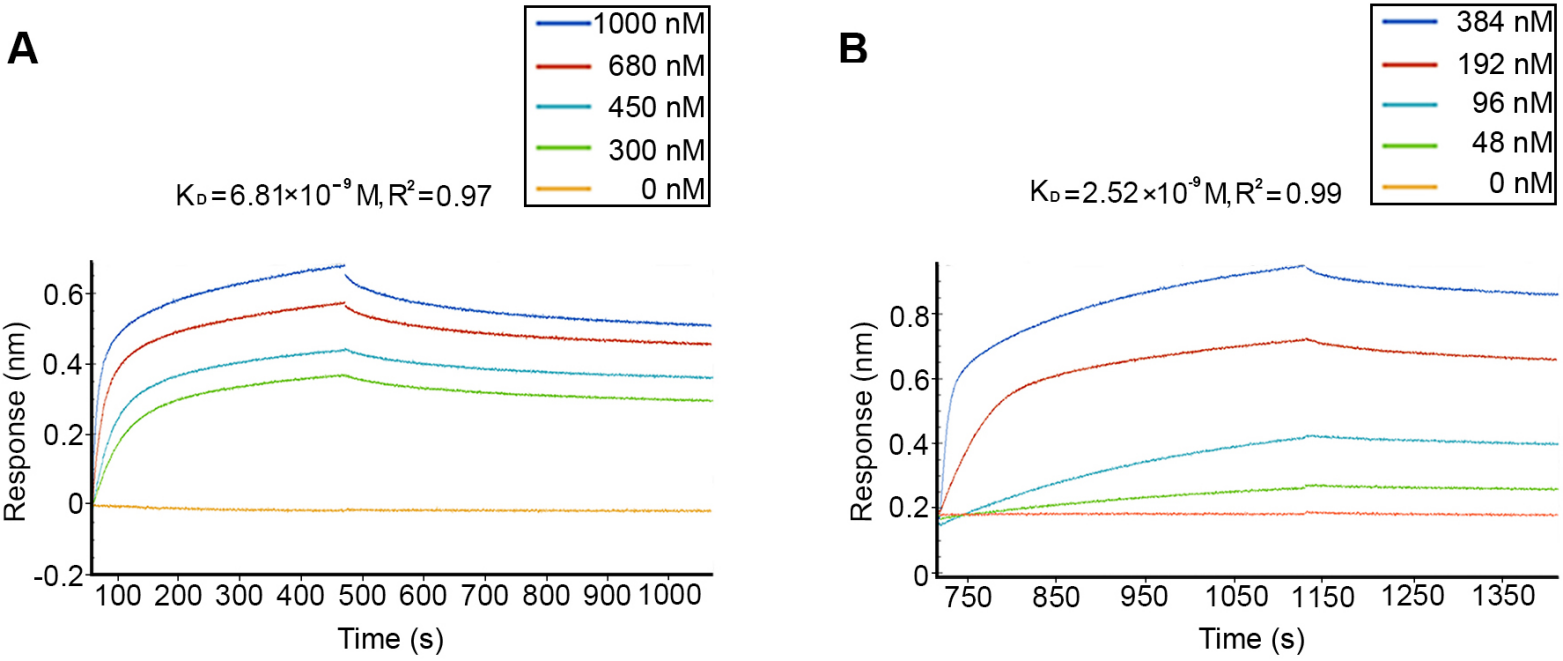
**The ForteBio Octet system was used to identify the interactions between rCHHBP and the intracellular and extracellular regions of rEsGLUT4.** (A) Intracellular region; (B) extracellular region. A sensorgram of rGLUT4 on streptavidin-coated biosensors binding to different concentrations of CHHBP is shown. rGLUT4 was labeled with EZ-Link™ NHS-LC-LC-Biotin. Saline buffer was used as a negative control. The binding affinity parameter K_D_ was calculated. R^2^ is the coefficient of determination for estimating the goodness of a curve fit reported by ForteBio Data Analysis Software 7.0.

We analyzed the interaction between the two hydrophilic regions of EsGLUT4 and CHHBP. As shown in Fig. 4, we screened five concentration ranges of the CHHBP samples to directly investigate the binding of CHHBP to biotin-EsGLUT4 intracellular and extracellular regions. For each process, four concentration gradients of CHHBP directly bound to the rEsGLUT4 protein, which were pre-labeled with biotin and immobilized onto the SA sensors, at a detectable level. The interactions between the intracellular and extracellular regions of EsGLUT4 and the CHHBP protein were the strongest when the sample concentrations of CHHBP were 1 µM and 0.38 µM, respectively (Fig. 4A,B). Crab saline buffer, which did not interact with EsGLUT4, was used as the negative control (yellow line). After the raw data were analyzed, the binding affinities of the rEsGlut4 intracellular and extracellular regions for rCHHBP were analyzed (R^2^=0.97 and R^2^=0.99, respectively), and the K_D_ values were measured (6.81×10^−9^ M and 2.52×10^−9^ M, respectively).

### EsGLUT4 and CHHBP colocalization in Hi5 insect cells

**Fig. 5.**
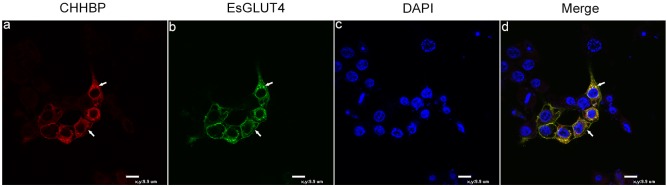
**Co-expression of CHHBP and EsGLUT4 in Hi5 insect cells.** pIZV5-CHHBP-Cherry and pIZV5-EsGLUT4-GFP were cotransfected into Hi5 insect cells using X-tremeGENE HP DNA Transfection reagent. The expression of the reporter genes Cherry and Enhanced Green Fluorescent Protein (EGFP), which represented CHHBP (red) and EsGLUT4 (green), respectively, was observed under a fluorescent confocal microscope at 24 h post-transfection (A,B). The nuclei were stained with 4′,6-diamidino-2-phenylindole (DAPI; blue) (C). Merged images showing overlap of CHHBP, EsGLUT4, and DAPI staining (D). Scale bar: 9.9 µm.

The cellular localization of EsGLUT4 and CHHBP was examined by co-expression, followed by confocal immunofluorescence microscopy. Over-expressed CHHBP in Hi5 insect cells was observed predominantly at the cell membrane and to a lesser extent in the cytoplasm (Fig. 5A). EsGLUT4 was expressed in cytosolic storage vesicles and at the cell membrane (Fig. 5B). Fig. 5D indicates that EsGLUT4 colocalized with CHHBP at the plasma membrane and in cytoplasmic storage vesicles under basal conditions.

### EsGLUT4 and CHHBP respond to EsCHH stimulation in Hi5 insect cells

**Fig. 6.**
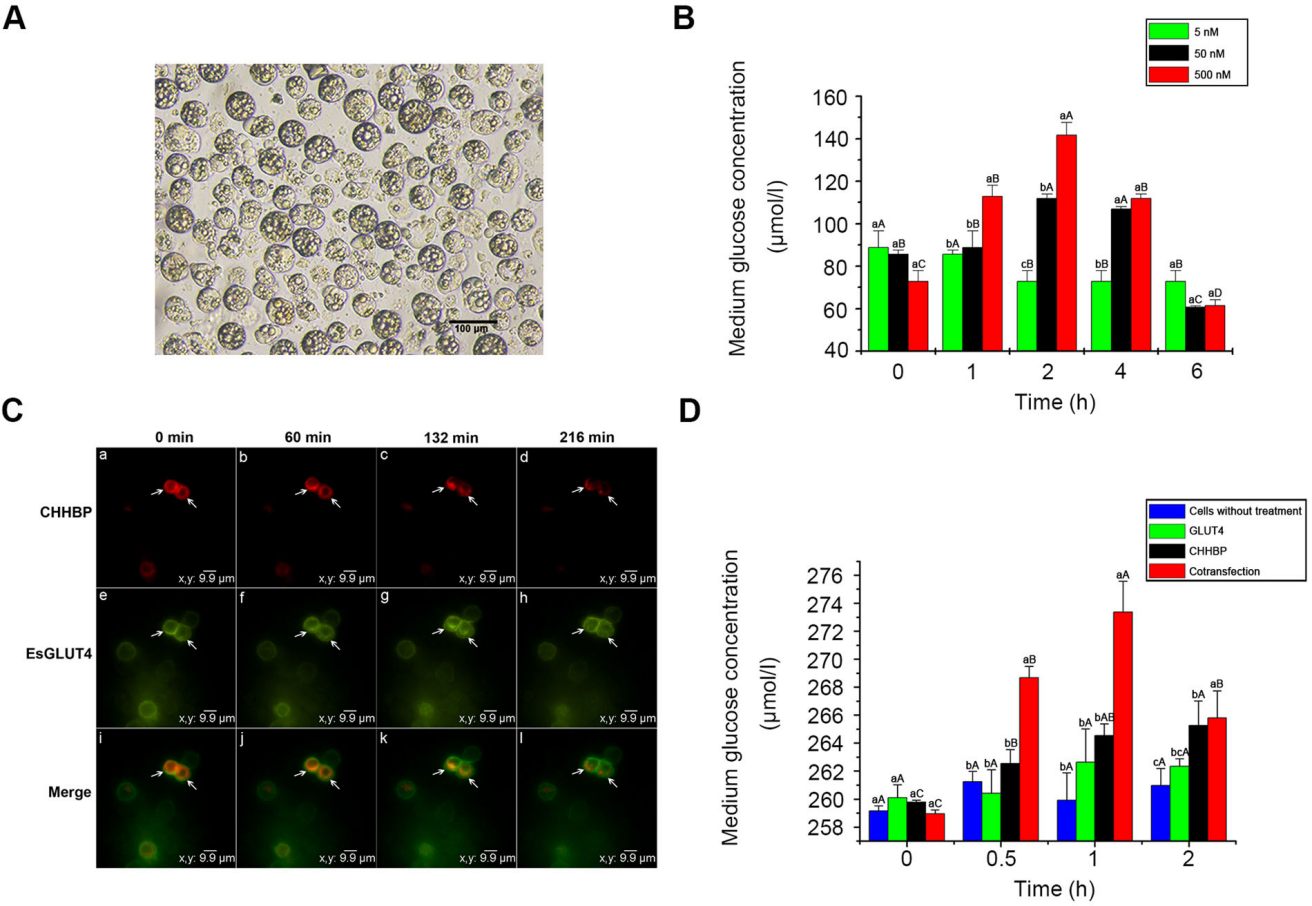
**The effects of EsCHH on hepatopancreatic cells.** (A) Hepatopancreatic cells from *E. sinensis* were cultured in medium for 2 days and examined under an inverted microscope. (B) The glucose concentration in the medium of cultured hepatopancreatic cells from *E. sinensis* after the addition of EsCHH at different concentrations. (C) EsCHH-induced EsGLUT4 and CHHBP showed different translocation patterns in Hi5 insect cells. CHHBP and EsGLUT4 were co-expressed and their translocation in Hi5 insect cells was monitored by live-cell imaging at different time points after stimulation with CHH. Detection of pIZV5-CHHBP-Cherry at 0, 60, 132, and 216 min (Ca-Cd). Detection of pIZV5-EsGLUT4-GFP at 0, 60, 132, and 216 min (Ce-Ch). Merged images show overlapping fluorescent signals for CHHBP, EsGLUT4, and DAPI (Ci-Cl). (D) The glucose concentration in the medium of cultured Hi5 insect cells at 0, 0.5, 1, and 2 h after stimulation with CHH. Cells were divided into four groups as follows: cells transfected with pIZV5-CHHBP-Cherry, cells transfected with pIZV5-EsGLUT4-GFP, cells cotransfected with pIZV5-CHHBP-Cherry and pIZV5-EsGLUT4-GFP, and cells without any treatment (control). In B and D, data denoted with different lowercase letters indicate significant differences within groups; the capital letters indicate significant differences between groups (*P*<0.05, one-way ANOVA followed by Duncan's analysis); mean±s.d.

To evaluate the effects of EsCHH on the glucose level, the media of cultured hepatopancreatic cells from *E. sinensis* were collected, and the glucose concentration was determined. The glucose level increased significantly at 1 and 2 h after the addition of EsCHH at a final concentration of 50 or 500 nM (Fig. 6B). The secreted glucose level was highest after the addition of 500 nM EsCHH. By contrast, there was no increase in the glucose level after the addition of 5 nM EsCHH. Therefore, 500 nM of EsCHH was used to investigate the CHH-induced localization of EsGLUT4 and CHHBP.

To investigate the CHH-induced localization of EsGLUT4 and CHHBP, pIZV5-CHHBP-Cherry and pIZV5-EsGLUT4-GFP were cotransfected into Hi5 insect cells, which were used instead of primary hepatopancreatic cells from *E. sinensis*, and EsCHH was added to the medium at a final concentration of 500 nM. Under basal conditions, pIZV5-CHHBP-Cherry and pIZV5-GLUT4-GFP colocalized at the cell membrane with some colocalization to cytoplasmic compartments (Fig. 6Ca,Ce,Ci). At 60 min after the addition of EsCHH, both EsGLUT4 and CHHBP began to appear in cytoplasmic vesicles (Fig. 6Cb,Cf,Cj). After 132 min, EsGLUT4 and CHHBP were mostly present in cytoplasmic vesicles (Fig. 6Cc,Cg,Ck). At 216 min after the addition of EsCHH, both EsGLUT4 and CHHBP translocated from the cytoplasmic vesicles to the plasma membrane (Fig. 6Cd,Ch,Cl). These results demonstrate for the first time that EsGLUT4 and CHHBP assemble within cytoplasmic vesicles and then translocate to the plasma membrane upon stimulation by EsCHH. A cartoon diagram was made to display how CHHBP and EsGLUT4 interacted with each other and transported glucose under the effect of CHH (Fig. S1).

We also measured the secreted glucose level in EsGLUT4- and CHHBP-cotransfected cells after EsCHH stimulation. Compared with the cells transfected with pIZV5-EsGLUT4-GFP alone, the glucose concentration in the medium increased significantly at 0.5, 1 h and 2 h after EsCHH stimulation in cells transfected with pIZV5-CHHBP-Cherry alone (black column) and in cotransfected cells (red column) (Fig. 6D)*.* Furthermore, the secreted glucose level was highest after EsCHH stimulation in the cotransfected group. By contrast, the glucose concentration did not change significantly after EsCHH stimulation in the other two groups (blue column and green column).

### Depletion of EsGLUT4 leads to a decreased blood glucose level and an increase in glycogen synthase expression *in vivo*

**Fig. 7.**
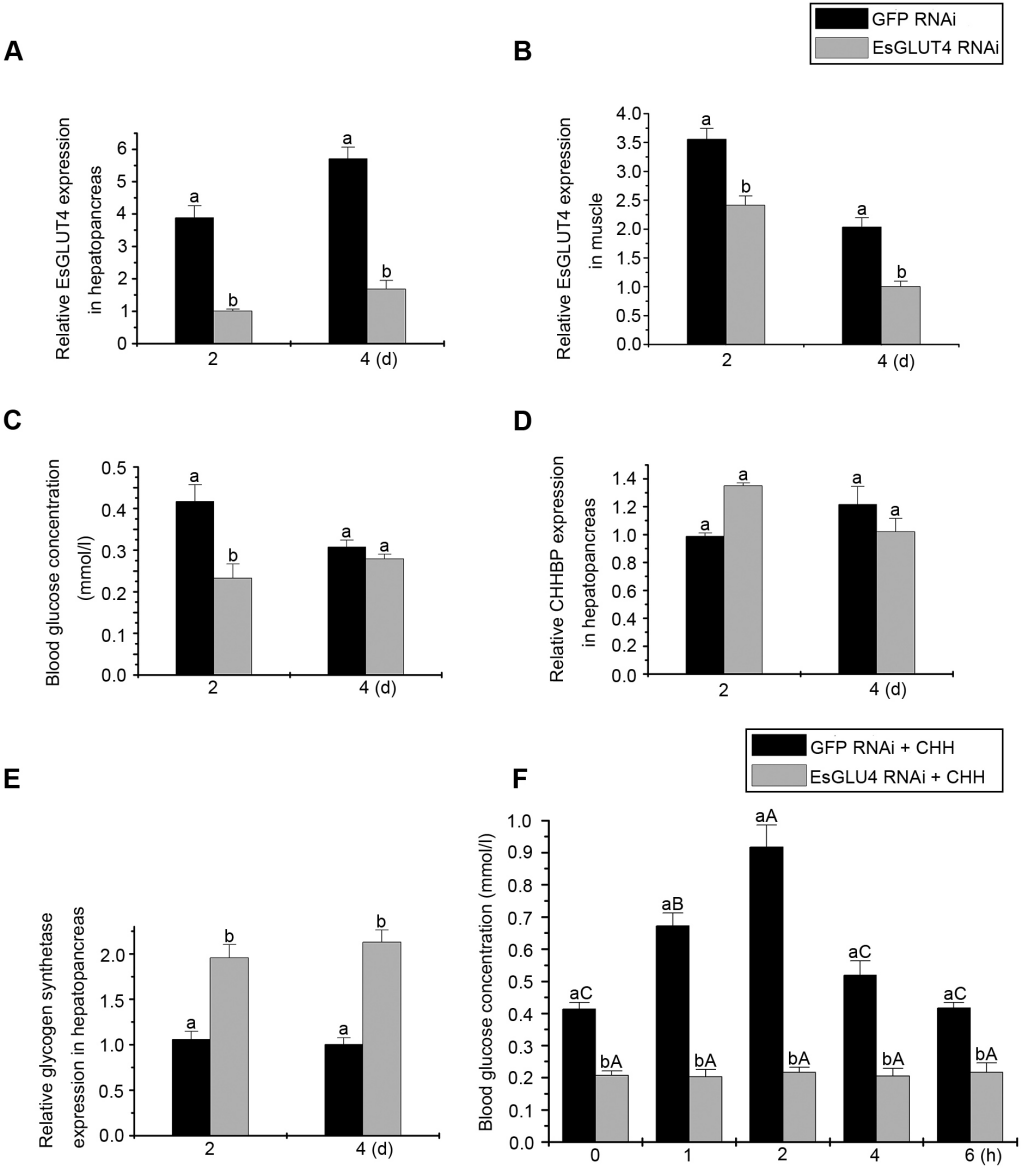
**Functional analysis of EsGLUT4 *in vivo*.** Crabs were injected with GFP dsRNA or EsGLUT4 dsRNA. The hepatopancreas tissues were collected to test gene knock-down efficiency. Transcript expression of EsGLUT4, CHHBP, and GS in hepatopancreas at 2 days and 4 days after dsRNA injection was assessed by quantitative real-time PCR (A,D,E). Transcript expression of EsGLUT4 in muscle was also assessed (B). β-actin was employed as an internal reference gene. Hemolymph glucose concentration of crabs after GFP RNAi or EsGLUT4 RNAi injection were measured (C). (F) Changes in glucose level in control (black bars) and EsGLUT4 RNAi group (gray bars) after injection of rCHH. Values are presented as means±s.d. (*n*=6). Data denoted with different lowercase letters indicate significant differences (*P*<0.05, Student's *t*-test following one-way ANOVA) between treatments.

Since EsGLUT4 translocated with CHHBP and contributed to the regulation of CHH in insect cells, we decided to further evaluate the function of EsGLUT4 by performing RNA inference in crabs (Fig. 7). GFP dsRNA (control) and EsGLUT4 dsRNA (test) were injected into crabs and qRT-PCR was used to detect the efficiency of dsRNA interference at 2 and 4 days after dsRNA injection. As shown in Fig. 7A, EsGLUT4 dsRNA knocked down most of the transcripts after 2 and 4 days of treatment. The amount of mRNA decreased to less than 10% of that of the control 2 days after EsGLUT4 dsRNA injection (2 days, Fig. 7A). Besides, EsGLUT4 transcripts were also reduced after 2 and 4 days of RNAi treatment in muscle (Fig. 7B).

Hemolymph was collected 2 days and 4 days after injection and its glucose concentration was analyzed using a glucose assay kit (Fig. 7C). Compared with the control group, the test group showed a significant decrease in hemolymph glucose concentration at 2 days after injection (Fig. 7C). However, since the glucose level also decreased in the control group, there was no significant difference between two groups at 4 days post-injection. Besides, in the control group, rCHH injection led to a gradually increased glucose level from 0 h to 2 h, followed by a gradually reduced glucose level. However, the already reduced glucose level induced by EsGLUT4 RNAi could not be rescued by injection of rCHH (Fig. 7F). The pairwise comparisons done following ANOVA indicated that there was a significant difference between the two groups at each time point.

To further investigate the effect of EsGLUT4 on glucose metabolism, we examined the mRNA expression levels of CHHBP and GS in the hepatopancreas by qPCR at 2 days and 4 days after knockdown of EsGLUT4. As shown in Fig. 7D, compared to the control group, the expression levels of CHHBP mRNA in the hepatopancreas showed no significant change at 2 and 4 days post-injection with dsRNA. The CHHBP mRNA expression level was constant compared with that of the dsGFP (control). Interestingly, the expression levels of GS mRNA increased significantly at 2 days and 4 days (Fig. 7E).

## DISCUSSION

We describe herein a novel assay to study EsGLUT4 trafficking upon stimulation with CHH, which regulates blood glucose levels and glucose metabolism throughout the crustacean life cycle. The sequence of EsGLUT4 was compared with human GLUT1-4. It turned out that EsGLUT4 shared 45% identity with human GLUT3 and GLUT4, and 44% identity with human GLUT1 (Fig. S2), but much less similarity with human GLUT2 (39%). BLASTp analysis revealed that the EsGLUT4 protein shared 58% sequence identity with the GLUT4 protein of *Phaedon cochleariae*, indicating that EsGLUT4 was highly homologous with GLUT4 in arthropoda (Fig. 2). Additionally, EsGLUT4 formed a sister group with GLUT4 proteins of the vertebrate class Osteichthyes that included sole (*Cynoglossus semilaevis*: XM_008321766.1), killifish (*Poecilia formosa*: XM_007551253.1), bream (*Neolamprologus brichardi*: XM_006807697.1), spotted gar (*Lepisosteus oculatus*: XM_006627433.1), and carp (*Astyanax mexicanus*: XM_007255725.1). In terms of function, both GLUT1 and -4 facilitate the transport of glucose in mammals, but they play distinct roles in different tissues. GLUT1 express mainly in erythrocytes while GLUT4 in muscle and adipose tissue. Based on the tissue expression analysis in *Eriocheir Sinensis* (Fig. S3), EsGLUT4 were mainly expressed in muscle but much less in hemolymph. We would be inclined to speculate the GLUT obtained from *Eriocheir Sinensis* is GLUT4. Several studies demonstrated that in mammals, GLUT4 interacts with flotillin upon insulin stimulation to transport glucose across the plasma membrane (Fecchi et al., 2006; Govers et al., 2004; Govers, 2014). To investigate the interaction between EsGLUT4 and the flotillin-like protein CHHBP *in vitro*, the ForteBio Octet system was used. GLUT4 is a membrane protein, possessing 12 transmembrane regions. Thus, it is difficult to express the entire GLUT4 protein using the prokaryotic expression system. To verify the interaction between EsGLUT4 and CHHBP, we successfully expressed and produced the recombinant intracellular and extracellular regions of GLUT4 (the two hydrophilic regions of EsGLUT4). These two regions were predicted to localize to the spherical surface of GLUT4 and to serve as the main interaction sites for EsGLUT4. Binding affinity analysis demonstrated that the hydrophilic regions of EsGLUT4 had strong binding affinity for CHHBP, which might play a crucial role in CHH-induced glucose release in crustaceans.

Because the hepatopancreas is also a target tissue of CHH (Chung et al., 2010; Katayama and Chung, 2009), and considering the difficulty of culturing primary muscle cells from *E. sinensis*, we used primary hepatopancreatic cells to measure the glucose level after CHH stimulation. To investigate the cellular localization of EsGLUT4 and CHHBP, we used the Hi5 insect cell line instead of *E. sinensis* primary cells. Although insect Hi5 cells were derived from ovary tissue as glucose utilizers, since CHH alone was reported to have minimal activity in insects (Meredith et al., 1996), it was used as a platform to perform the *in vitro* expression, without cross effects between species. In addition, we tried to deliver the recombinant plasmid into primary blood cells and hepatopancreatic cells from *E. sinensis* using several methods, but without success. The possible reasons for this failure might be that the primary cells are highly differentiated and that gene delivery was inefficient at the cellular level, which is a major obstacle for crustacean research. Furthermore, few promoters can initiate exogenous gene expression in primary cells (Li et al., 2011). Therefore, we chose an insect cell line to complete the study.

Fecchi et al. (2006) showed that GLUT4 is present in flotillin-1-containing membranes of skeletal muscle cells from mammals, forming a stable complex with flotillin-1. We showed that EsGLUT4 colocalized with CHHBP at the plasma membrane and in the cytoplasm of Hi5 insect cells (Fig. 5). With few exceptions, there were some large vesicles that seem to contain only CHHBP. Since the expression efficiency of these two plasmids co-transfected into cells might be different, we speculated that different numbers of them did not result in one-to-one binding. We demonstrated for the first time that CHH increased GLUT4-dependent glucose trafficking in Hi5 insect cells (Fig. 6C). We also described an assay to study EsGLUT4 trafficking under CHH stimulation and showed that unlike insulin-responsive GLUT4 trafficking in mammals, EsGLUT4 and CHHBP transiently localized to cytoplasmic vesicles (Fig. 6Cc,Cg, Ck), subsequently translocating to the plasma membrane upon CHH stimulation in Hi5 insect cells (Fig. 6Cd,Ch,Cl). Moreover, CHH promoted glucose release from hepatopancreatic cells from *E. sinensis* (Fig. 6B), and the glucose level in the medium of EsGLUT4- and CHHBP-cotransfected cells increased significantly after the addition of CHH (Fig. 6D, red column). In addition, the results of the glucose assay showed that EsCHH can promote glucose release from Hi5 cells transfected with pIZV5-CHHBP-Cherry alone or cotransfected with EsGLUT4 and CHHBP (Fig. 6D, black column and red column)*.* However, the secreted glucose level was highest after EsCHH stimulation in the cotransfected group. This could be because Hi5 cells contain proteins that are homologous to GLUT4 and binding of CHHBP to EsCHH mobilized these homologous molecules to transport glucose. Based on the result of live-cell imaging and glucose assay, Fig. S1 summarizes a tentative cartoon model in highly schematic form, indicating how CHHBP might interact with EsGLUT4 under the signal of CHH. After cotransfection into Hi5 cells, CHHBP mainly localized on the cell membrane, while EsGLUT4 localized in the cytoplasm (Fig. S1A). Shortly after adding CHH into the medium, CHHBP, as the receptor, bound with CHH. Next, some of CHHBP moved to the cytoplasm and interacted with EsGLUT4, forming a complex (Fig. S1B). Then the CHHBP-EsGLUT4 complex carried the glucose on and moved towards the membrane, followed by its merge into the membrane, releasing the glucose out of the cell (Fig. S1C). Insect cells are used here as a platform to show the relationship network among three exogenous molecules, besides, there are no gene homologous to CHH and CHHBP in this system. EsGLUT4, as a foreign protein over expressed in these cells, might function in a different way to the endogenous glucose transporters. Considering all the above factors, we conclude that EsGLUT4 might form a protein complex with CHHBP to regulate CHH-stimulated glucose release in *E. sinensis*.

In recent years, considerable effort has been expended in investigating members of the human glucose transporter family, but comparatively little research has been expended in investigating glucose transporters in crustaceans. A D-glucose transporter (SGLT) was previously found in the decapod crustacean hepatopancreas (Ahearn et al., 1985; Sterling et al., 2009; Verri et al., 2001). However, little work has been reported on the role of GLUT4 in the glucose transport process in crustaceans. To identify the function of EsGLUT4, we examined changes in blood glucose concentrations and analyzed the effect of EsGLUT4 silencing on the expression levels of downstream molecules. As shown in Fig. 7, at 2 days post-injection, there was a significant decrease in the hemolymph glucose concentration in the group injected with EsGLUT4 dsRNA, compared with the glucose concentration in GFP dsRNA-treated (control) crabs (*P*<0.05) (Fig. 7C), suggesting that EsGLUT4 is involved in the physiological process of glucose transport. The decline in the expression of EsGLUT4 in muscle due to RNAi may have weakened the ability of muscle cells to transfer glucose inside. However, although the mechanism of how CHH acts on muscle remains unclear, according to our long-term study of CHH receptor in hepatopancreas (CHHBP) and its regulatory pathway, EsGLUT4 RNAi blocks the signal pathway downstream of CHH, and then the role of CHH hyperglycemia is decreased. In the following fasting process, the degree of decreased hyperglycemic effect maybe exceed the extent of glucose intake by muscle cells, leading to the final decrease in the hemolymph glucose concentration. In addition, since GS plays an important role in glycogen metabolism in liver and muscle (Sedlmeier, 1982) and EsGLUT4 was proved to be involved in this physiological process, we also analyzed the relative expression levels of CHHBP and GS after EsGLUT4 knockdown. The results showed no significant change in the expression levels of CHHBP mRNA in the hepatopancreas at 2 and 4 days after injection of EsGLUT4 dsRNA (Fig. 7D), but the expression levels of GS mRNA increased significantly after 2 and 4 days (Fig. 7E). We speculate that CHHBP acts as an upstream signaling molecule in the EsGLUT4 pathway (Fig. S1). Furthermore, GLUT4 RNAi interrupted signal transduction 39% downstream from CHH (Fig. 7E). The expression of glycogen synthase (GS), which catalyzes a key step in glycogen synthesis, increased considerably after the injection of EsGLUT4 dsRNA, probably to speed up the conversion of glucose into glycogen. This would be consistent with a previous observation showing glycogen accumulation in hepatopancreas cells treated with CHHBP RNAi (Li et al., 2016). Combined with the *in vitro* result shown in Fig. 6, we confirmed that EsGLUT4 could transport glucose and play a crucial role in glucose metabolism in *Eriocheir sinensis*. In conclusion, our results indicate that EsGLUT4 colocalizes with CHHBP and contributes to CHH-dependent signal transduction in crustaceans, which may provide a theoretical basis for studying the mechanism of glucose transport and metabolism responsible for GLUT4 translocation under the control of CHH in crustaceans.

## MATERIALS AND METHODS

### Animals

Chinese mitten crabs (*E. sinensis*) approximately 15±5 g (*n*=10) in weight were purchased from a local market in Tianjin, China. The crabs were maintained in aerated fresh water at 24–26°C for one week before processing. Only healthy crabs without any pathological signs were used in the experiments. All animal studies were performed with the approval of Tianjin Normal University Animal Ethics Committee.

### RNA extraction and cDNA synthesis

First-strand cDNA was synthesized from 2 µg of high-quality DNase I-treated (Promega, Madison, WI, USA) total RNA with an AOLP 3′-adaptor primer (Table S1) (Promega) and a 5′-adaptor GeneRacer™ Oligo primer (Table S1) (Invitrogen, Carlsbad, CA, USA) according to the manufacturers' instructions. The reaction was performed at 37°C for 30 min, 70°C for 5 min, and 42°C for 1 h, followed by incubation on ice for 2 min and termination by heating at 95°C for 5 min. To clone full-length EsGLUT4, the synthesized cDNA was used for the rapid amplification of cDNA ends (RACE).

### Cloning of full-length EsGLUT4 cDNA

To obtain the N-terminal fragment of the ORF and the entire 5′-untranslated region of EsGLUT4, 5′-RACE was performed with specific primers (GLUT4 5′ R1 and R2, Table S1) using the GeneRacer™ kit (Invitrogen). To clone the entire 3′-untranslated region, 3′-RACE was performed using specific primers (GLUT4 3′ F1 and F2, Table S1). All gene-specific primers were designed based on the transcriptome, which included a C-terminal fragment of the GLUT4 ORF. Polymerase chain reaction (PCR) was performed using the cDNAs synthesized from the hepatopancreas of *E. sinensis* as a template, and the PCR products were separated by electrophoresis on a 1% agarose gel. The band with the expected size was excised, purified, and cloned into the pMD18-T vector for nucleotide sequencing to obtain the entire 5′- and 3′-untranslated regions and the N-terminal fragment of the ORF. The full-length EsGLUT4 cDNA was spliced with DNA-Star 5.01, and specific primers (GLUT4 F and R, Table S1) were designed to obtain the full-length EsGLUT4 cDNA.

### Analysis of nucleotide and amino acid sequences

The nucleotide sequence was blasted against the GenBank database at the National Center for Biotechnology Information (www.ncbi.nlm.nih.gov/blast) using the BLASTx algorithm to identify the encoded protein. Protein motif features were analyzed using the simple modular architecture research tool (SMART) program (www.smart.embl-heidelberg.de), the transmembrane helices were predicted by TMHMM (www.expasy.org), and the theoretical isoelectric point (pI) and molecular weight (Mw) were calculated with an online software program (http://web.expasy.org/compute_pi/).

The multiple sequence alignment was created with ClustalW (Aiyar, 2000; Larkin et al., 2007). A neighbor-joining (NJ) tree was constructed based on the sequence alignment using the NJ algorithm in the MEGA4.0 software package (Tamura et al., 2007). Bootstrap trials were replicated 1000 times to derive confidence values for the phylogenetic analysis.

### Recombinant plasmid construction of the intracellular and extracellular regions of EsGLUT4

The GLUT4 DNA fragments encoding the extracellular region between TM1 (transmembrane region 1) and TM2 and the intracellular region between TM6 and TM7 were amplified using two primer pairs (pET GLUT4 Ex F/R and pET GLUT4 In F/R, respectively, Table S1). The amplified fragments were digested with NdeI and HindIII and then inserted into the pET-28a vector, which had been digested with the same enzymes. The recombinant intracellular region plasmid and the extracellular region plasmid were transformed into competent Rosetta-gami (DE3) cells for recombinant protein expression. Positive clones were screened by PCR and confirmed by nucleotide sequencing.

### Expression and purification of the recombinant CHH, EsGLUT4 and CHHBP proteins

Rosetta-gami (DE3) cells were transformed with the recombinant CHH, EsGLUT4 intracellular and extracellular regions, CHHBP expression plasmid. Cultures of the transformants grown in 300 ml of LB medium containing antibiotics at 37°C with shaking at 220 rpm until the culture OD_600_ reached 0.6. Then, 1 mM L-1 isopropyl-b-D-thiogalactoside (IPTG) was added to the medium under the same conditions. Bacterial pellets were collected by centrifugation at 4500 rpm, 4°C and then broken by Ultrasonic cell Disruption System (Scientz Biotechnology, China) after resuspended with fragmentation buffer (50 mM Tris–HCl, 100 mM NaCl, 2 mM EDTA, 0.5% TritionX-100, 1 mg/ml Lysozyme). The recombinant CHH, EsGLUT4 intracellular and extracellular regions and CHHBP were located in inclusion bodies. The inclusion bodies purified by affinity chromatography with Ni–nitrilotriacetic acid (NTA) agarose (GE) under denaturing (8 M urea) conditions. The resultant proteins were detected by SDS-PAGE. Protein refolding was achieved by dialyzing the solution against refolding buffer (5 mM L-cysteine and 50 mM Tris) containing urea (8, 6, 4, 3, 2, and 1 M) progressively; each dialysis step was performed for at least 4-6 h at 4°C. Last, the refolded CHH, EsGLUT4 intracellular and extracellular regions and CHHBP protein were dialyzed against physiological saline for crab (205.13 mM NaCl, 5.37 mM KCl, 13.51 mM CaCl_2_, 2.61 mM MgCl_2_·6H_2_0, 2.39 mM NaHCO_3_, and 13.93 mM HEPES), an improved formula (Sashikumar and Desai, 2008) according to Van Harreveld's, before use.

### ForteBio Octet system assay

The ForteBio Octet (ForteBio Inc., USA) system was used to determine the interactions between rCHHBP (recombinant CHHBP) and the rEsGLUT4 (recombinant EsGLUT4) intracellular and extracellular regions. Experiments were performed at room temperature following the ForteBio manufacturer's protocol. First, the rEsGLUT4 intracellular and extracellular regions were labeled with biotin using EZ-Link™ NHS-LC-LC-Biotin (Thermo Scientific, USA) to enable binding to the streptavidin biosensor. In detail, the appropriate volume of biotin was dissolved in dimethyl sulfoxide (DMSO) to a final concentration of 10 mM. Then it was added to the EsGLUT4 intracellular and extracellular region solutions (dissolved in physiological saline for crab). The reaction was incubated at room temperature for 30 min. Excess non-reacted biotin was removed using Zeba™ Spin Desalting Columns (Thermo Scientific, USA). The biotin-labeled and desalted rEsGLUT4 intracellular and extracellular region proteins were dissolved in saline buffer at a concentration of 50 µg/ml for loading into each well of an Octet 96-well microplate and were then immobilized onto the streptavidin (SA) sensors. Four serial concentrations of the CHHBP sample and a negative control (saline buffer) were used for each experiment. The Octet assay protocol was performed as follows: baseline (saline buffer) for 60 s, loading (50 µg/ml biotinylated and desalted rEsGLUT4 intracellular and extracellular region proteins) for 600 s, baseline 2 for 60 s, association of test sera for 300 s, disassociation for 600 s, and finally, neutralization and regeneration for 5 s for 3 cycles. The highest CHHBP sample concentrations were 1 µM and 0.38 µM. The detector could track the interaction between CHHBP and the EsGLUT4 intracellular and extracellular regions on the SA sensors throughout the process. Dissociation constants were calculated from the raw data using the analysis software accompanying the Octet Red96 Data analysis system (version 8.0, ForteBio).

### Cell culture and plasmid constructs

Crabs were anesthetized on ice for 5 min and washed with sterile water. Then, the hepatopancreas was dissected and rinsed twice with PBS containing antibiotics (1.54 mM KH_2_PO_4_, 155.17 mM NaCl, 2.71 mM Na_2_HPO_4_-7H_2_O, 100 μg/ml penicillin, and 100 μg/ml streptomycin, Thermo Fisher Scientific, USA). After the tissue was minced into 1-3 mm pieces, it was centrifuged at 800 ***g*** for 5 min. Primary hepatopancreas cells were collected through filtration with a cell strainer and centrifugation and then cultured in L-15 medium (catalog no. 21083027, containing 10% fetal bovine serum, 100 μg/ml penicillin, and 100 μg/ml streptomycin, Thermo Fisher Scientific, USA) at 26°C with 5% CO_2_. Hi5 insect cells were cultured at 27°C without CO_2_ in Grace's medium (catalog no. 11605094, Thermo Fisher Scientific, USA) supplemented with 10% fetal bovine serum, penicillin (100 μg/ml) and streptomycin (100 μg/ml). All cell culture and transfection reagents were obtained from Gibco, Invitrogen.

The red fluorescent protein (mCherry) and green fluorescent protein (GFP) sequence was amplified from the pmCherry C1 and EGFP-N1 vector, and then cloned into pIZV5-His (Invitrogen, Carlsbad, California), to generate pIZV5-Cherry and pIZV5-GFP. Two pairs of primers (pIZ EsGLUT4-GFP F/R and pIZ CHHBP-Cherry F/R, Table S1) were designed to amplify the ORF encoding EsGLUT4 and CHHBP. The recombinant plasmids pIZV5-EsGLUT4-GFP and pIZV5-CHHBP-Cherry were transformed into competent DH5α cells for nucleotide sequencing and then extracted using a Qiagen^®^ plasmid midi kit (Qiagen).

### Cotransfection of EsGLUT4 with CHHBP

Hi5 cells were cultured in 12-well plates at 27°C with 1 ml of Grace's medium (catalog no. 11605094, Thermo Fisher Scientific, USA) containing 10% fetal bovine serum. The cells were cotransfected with the pIZV5-CHHBP-Cherry and pIZV5-GLUT4-GFP plasmids at a 2:1 ratio following the Roche X-tremeGENE HP DNA Transfection Reagent protocol (Roche, Switzerland). A total of 2 μg (1.33 μg:0.67 μg, respectively) of the recombinant plasmids pIZV5-CHHBP-Cherry and pIZV5-EsGLUT4-GFP was mixed well with 3 μl of the Roche X-tremeGENE HP DNA transfection reagent and incubated for 30 min at 25°C before being added to each well. Protein expression was measured by confocal immunofluorescence microscopy (Nikon Eclipse 90i, Japan) after 18-72 h of incubation under suitable culture conditions. The artwork was created by Adobe Photoshop (Adobe Systems).

### EsCHH treatment of primary hepatopancreatic cells of *E. sinensis* and measurement of glucose levels

The primary hepatopancreatic cells of *E. sinensis* were cultured as described above and divided into three groups according to the concentration of CHH to be added. Prior to the measurement of the glucose level, the primary cell culture medium was replaced with L-15 medium (containing 10% fetal bovine serum, 100 μg/ml penicillin, 100 μg/ml streptomycin), followed by the addition of EsCHH at a final concentration of 5, 50, or 500 nM. Thereafter, the hepatopancreatic cells were stimulated with CHH for 0 (control), 1, 2, 4, and 6 h. In each group by EsCHH concentration, there were six repeats (wells) to be tested at each time point. The cell culture medium was collected and its glucose concentration was determined using a glucose assay kit (Rongsheng Biotech Co., Shanghai, China) according to the manufacturer's instructions. Origin 8.0 mapping software (Origin Lab, USA) was used to analyze the data. Multiple group comparisons were conducted by one-way ANOVA followed by Duncan's analysis using SPSS software version 17.0 (IBM, USA). The data are presented as the means±standard deviation (s.d.) (*n*=6). Differences were considered significant at *P*<0.05.

### EsCHH stimulation of Hi5 cells

Approximately 5×10^6^ Hi5 insect cells were seeded into each well of a 12-well culture plate. All cells were divided into four groups: cells transfected with pIZV5-CHHBP-Cherry alone, cells transfected with pIZV5-EsGLUT4-GFP alone, cells cotransfected with pIZV5-CHHBP-Cherry and pIZV5-EsGLUT4-GFP, and untransfected cells (controls). Each group had three replicates. At 24 h post-transfection, the fluorescence of each group was observed using an Olympus microscope system. To investigate the effect of CHH on each group of cells, EsCHH was added to a final concentration of 500 nM. Then, the medium were collected at 0 min, 30 min, 1 h, and 2 h after EsCHH stimulation to evaluate glucose concentrations. Glucose levels were determined as described previously.

### Live-cell imaging

Hi5 cells were cultured in 35-mm vitreous plates at 27°C with 1.5 ml of Grace's medium containing 10% fetal bovine serum. pIZV5-CHHBP-Cherry and pIZV5-EsGLUT4-GFP were cotransfected in a 2:1 ratio into Hi5 insect cells. A live-cell imaging system (Nikon Confacal Microscope A1R, Japan) was used to monitor the responses of CHHBP and EsGLUT4 to EsCHH stimulation in the Hi5 insect cells. Brightness and contrast of images were adjusted by the software of Image J (National Institutes of Health) and the artwork was created by Adobe Photoshop (Adobe Systems).

### Synthesis of double-stranded RNAs and injection

A primer pair (dsGLUT4 F and dsGLUT4 R, Table S1) was designed to amplify a fragment of the *EsGLUT4* ORF. The amplified fragment was digested with Xba I and EcoR II and then inserted into the pETT7 vector. The recombinant pETT7-GLUT4 plasmid was transformed into competent *E. coli* DH5α cells for DNA sequencing. The sequenced plasmid was then transformed into *E. coli* HT115 cells, and the dsRNA was purified as described by Yodmuang et al. (2006).

For RNAi experiments, crabs were acclimated in a culture tank overnight prior to injection. Then, approximately 10 μg of double-stranded RNA of EsGLUT4 (EsGLUT4 dsRNA) and green fluorescent protein (GFP dsRNA, as a control) dissolved in 20 μl of sterile water were injected into each crab. At 2 and 4 days after injection, the hepatopancreases and muscle were harvested and crushed in liquid nitrogen for RNA extraction using TRIzol reagent (Invitrogen). cDNA was synthesized from the total RNA using M-MLV reverse transcriptase (Promega). The relative expression of *EsGLUT4*, CHHBP, and GS were determined by quantitative real-time PCR (qRT-PCR) using an ABI 7500 cycler and FastStart Universal SYBR Green Master Mix (Roche, Switzerland) according to the manufacturer's instructions. β-actin served as the internal reference gene. Primer pairs used for the qRT-PCR detection were dsGLUT4 F/R, Q-GLUT4 F/R, Q-CHHBP F/R, Q-GS F/R, and β-actin F/R, respectively (Table S1). The cycling conditions for qRT-PCRs were as follows: initial denaturation at 95°C for 10 min, 40 cycles at 95°C for 15 s, and 60 s at 57°C, 58°C, and 60°C, followed by dissociation curve analysis from 55°C to 95°C at 5 s increments of 0.5°C. The relative expression level was determined by the comparative 2^−ΔΔCt^ quantification method (Livak and Schmittgen, 2001). The data are presented as the means±s.d. from six copies of experiments. The origin 8.0 mapping software was used to process the data (Origin Lab) and data denoted with different lowercase letters indicate significant differences among intragroups (*P*<0.05). Student's *t*-test was used to compare results from two groups following one-way ANOVA.

At 2 and 4 days after injection of dsRNA, haemolymph was collected with a 1 ml syringe and immediately diluted with an equal volume of anti-coagulant buffer (0.3 M NaCl, 20 mM trisodium citrate, 26 mM citric acid, and 1 mM EDTA). The blood glucose level was measured was determined as described previously. To detect the glucose level on the effect of RNAi, crabs (15±5 g) were injected with 1.5 μg rCHH each at 2 days after dsRNA injection. Haemolymph samples were extracted at 0 h, 1 h, 2 h, 4 h and 6 h after the injection and analyzed for their glucose concentration.
